# Ability of artificial intelligence to diagnose coronary artery stenosis using hybrid images of coronary computed tomography angiography and myocardial perfusion SPECT

**DOI:** 10.1186/s41824-019-0052-8

**Published:** 2019-03-18

**Authors:** Hiroto Yoneyama, Kenichi Nakajima, Junichi Taki, Hiroshi Wakabayashi, Shinro Matsuo, Takahiro Konishi, Koichi Okuda, Takayuki Shibutani, Masahisa Onoguchi, Seigo Kinuya

**Affiliations:** 1 Department of Radiological Technology, Kanazawa University Hospital, 13-1 Takara-machi, Kanazawa, 920-8641 Japan; 2 Department of Nuclear Medicine, Kanazawa University Hospital, Kanazawa, Japan; 3 Department of Physics, Kanazawa Medical University, Kahoku, Japan; 4 Department of Quantum Medical Technology, Institute of Medical, Pharmaceutical and Health Sciences, Kanazawa University, Kanazawa, Japan

**Keywords:** Cardiac evaluation, Nuclear cardiology, Ischemia, Computer-aided diagnosis, Neural network

## Abstract

**Background:**

Detecting culprit coronary arteries in patients with ischemia using only myocardial perfusion single-photon emission computed tomography (SPECT) can be challenging. This study aimed to improve the detection of culprit regions using an artificial neural network (ANN) to analyze hybrid images of coronary computed tomography angiography (CCTA) and myocardial perfusion SPECT.

**Methods:**

This study enrolled 59 patients with stable coronary artery disease (CAD) who had been assessed by coronary angiography within 60 days of myocardial perfusion SPECT. Two nuclear medicine physicians interpreted the myocardial perfusion SPECT and hybrid images with four grades of confidence, then drew regions on polar maps to identify culprit coronary arteries. The gold standard was determined by the consensus of two other nuclear cardiology specialist based on coronary angiography findings and clinical information. The ability to detect culprit coronary arteries was compared among experienced nuclear cardiologists and the ANN. Receiver operating characteristics (ROC) curves were analyzed and areas under the ROC curves (AUC) were determined.

**Results:**

Using hybrid images, observer A detected CAD in the right (RCA), left anterior descending (LAD), and left circumflex (LCX) coronary arteries with 83.6%, 89.3%, and 94.4% accuracy, respectively and observer B did so with 72.9%, 84.2%, and 89.3%, respectively. The ANN was 79.1%, 89.8%, and 89.3% accurate for each coronary artery. Diagnostic accuracy was comparable between the ANN and experienced nuclear medicine physicians. The AUC was significantly improved using hybrid images in the RCA region (observer A: from 0.715 to 0.835, *p* = 0.0031; observer B: from 0.771 to 0.843, *p* = 0.042). To detect culprit coronary arteries in perfusion defects of the inferior wall without using hybrid images was problematic because the perfused areas of the LCX and RCA varied among individuals.

**Conclusions:**

Hybrid images of CCTA and myocardial perfusion SPECT are useful for detecting culprit coronary arteries. Diagnoses using artificial intelligence are comparable to that by nuclear medicine physicians.

## Background

Myocardial perfusion imaging (MPI) is widely used to noninvasively assess reversible myocardial ischemia (Henzlova et al. 2016; Matsumoto and Hirayama 2017; Kiso et al. 2017; Matsuda and Takeishi 2016). Hachmovitch et al. reported that 10% ischemia of the left ventricle can be used as a guide to effective coronary revascularization, and a Japanese multicenter study also demonstrated that ≥ 5% of ischemic reduction led the improvement of patient outcome (Hachmovitch et al. 2003; Shaw et al. 2008; Nanasato et al. 2018). While importance of the assessment of ischemia is included in nuclear cardiology guidelines at present, all the institutions do not necessarily have nuclear cardiology specialists. Artificial intelligence (AI) has also been applied to nuclear cardiology and might aid in the interpretation of single-photon emission computed tomography (SPECT) images (Arsanjani et al. 2013a; Motowani et al. 2016; Arsanjani et al. 2013b; Arsanjani et al. 2015; Guner et al. 2010; Nakajima et al. 2018; Nakajima et al. 2017; Nakajima et al. 2015; Johansson et al. 2014). Since the AI can identify possible abnormal regions with a degree of probability, it should be important to clarify how we can use AI for the clinical practice. On the other hand, although coronary angiography is regarded as the gold standard for a diagnosis of coronary artery disease (CAD), it is invasive and not the first choice for assessing patients. Thus, coronary computed tomography angiography (CCTA) is rapidly gaining clinical acceptance (Kiriyama et al. 2018), and it complements myocardial perfusion SPECT (MP-SPECT) in the assessment of CAD (Levine et al. 2011; Sato et al. 2015). Kirisli et al. reported that hybrid images of MP-SPECT and CCTA offered the additional diagnostic benefit of allowing myocardial perfusion defect correlations with corresponding coronary arteries (Kirisli et al. 2014). Furthermore, Slomka et al. reported that CTA-guided MPI analysis correctly identified stenotic lesions that had been confirmed by invasive angiography, particularly in the right coronary (RCA) and left circumflex (LCX) arteries, whereas CTA alone and MPI unaided by CTA did not (Slomka et al. 2009). Several detailed studies have investigated the clinical usefulness of hybrid images. However, none have focused on interpreting hybrid images using cardioREPO/EXINI Heart software (FUJIFILM Toyama Chemical Co. Ltd., Tokyo, Japan; EXINI Diagnostics AB, Lund, Sweden). The present study aimed to determine the diagnostic ability of this software with an AI component to detect culprit coronary arteries on hybrid images.

## Methods

### Patient population

Table 1 shows the patient characteristics. The study group comprised 59 patients with stable CAD who had been assessed by coronary angiography within 60 days of MP-SPECT. All patients underwent stress MPI using adenosine at an infusion rate of 120 μg/kg/min for 6 min. At 3 min after starting the adenosine infusion, 250–370 MBq of ^99m^Tc-sestamibi (MIBI) (FUJIFILM Toyama Chemical Co. Ltd.) or tetrofosmin (Nihon Medi-Physics Co. Ltd., Tokyo, Japan) was injected. The rest of the study included 740–1110 MBq of ^99m^Tc-tetrofosmin or MIBI. The patients were assessed by SPECT approximately 60 min after injection. All patients underwent a single-day stress-rest protocol.

**Table 1:** Demographics of patients assessed by MP-SPECT and invasive coronary artery angiography

	Mean ± SD or number (*n*)
Age (year)	70.2 ± 10.6
Gender (M vs. F)	43 vs. 14
Height (cm)	162.5 ± 10.4
Weight (kg)	61.9 ± 13.6
Body mass index (kg/m^2^)	23.2 ± 3.4
Hypertension	48 (81.4%)
Diabetes mellitus	42 (71.2%)
Dyslipidemia	34 (57.6%)
eGFR (mL/min/1.73 m^2^)	61.6 ± 27.0
No significant stenosis	20 (33.9%)
1-vessel disease	20 (33.9%)
2-vessel disease	11 (18.6%)
3-vessel disease	8 (13.6%)
Regions of vessels with ≥ 50% stenosis	
LAD	26 (44.1%)
LCX	15 (25.4%)
RCA	19 (32.2%)
Regions of vessels with ≥ 75% stenosis	
LAD	18 (30.5%)
LCX	11 (18.6%)
RCA	15 (25.4%)
SSS (LAD, LCX, RCA, total)	2.3 ± 2.8, 3.1 ± 3.4, 2.7 ± 3.1, 7.8 ± 6.8
SRS (LAD, LCX, RCA, total)	0.92 ± 1.7, 2.0 ± 2.8, 1.7 ± 2.8, 4.3 ± 5.4
SDS (LAD, LCX, RCA, total)	1.2 ± 1.7, 1.0 ± 1.3, 1.1 ± 1.5, 3.4 ± 3.1
EDV (mL) at rest	98.4 ± 60.3
ESV (mL) at rest	50.3 ± 54.0
LVEF (%) at rest	57.6 ± 17.2

Data are shown as means ± SD or numbers (*n*). *EDV* end-diastolic volume, *eGFR* estimated glomerular filtration rate, *ESV* end-systolic volume, *F* female, *LAD* left anterior descending coronary artery, *LCX* left circumflex coronary artery, *LVEF* left ventricular ejection fraction, *M* male, *MP* myocardial perfusion, *RCA* right coronary artery, *SD* standard deviation, *SDS* summed difference score, *SPECT* single photon emission computed tomography, *SRS* summed rest score, *SSS* summed stress score

### MP-SPECT images

#### Acquisition

Patients were assessed by MP-SPECT using a Symbia T6 SPECT-CT system (Siemens, Erlangen, Germany; Siemens Japan, Tokyo, Japan). The equipment consisted of a dual-head gamma camera in 180° geometry equipped with a low-energy, high-resolution (LEHR) collimator. The SPECT data were acquired in a 64 × 64 matrix using the following parameters: zoom, × 1.45; pixel size, 6.6 mm, duration, and 35 s per projection for a total acquisition period of 20 min. We obtained a total of 60 frames, at 6° per step over 360°. The cardiac cycle was divided into 16 frames for electrocardiography (ECG) gating. R-R intervals that averaged ± 25% on the ECG monitor were accepted for gating. The energy was centered at 140 keV, with a 15% window for SPECT/CT imaging. Scatter and attenuation were not corrected. The detectors were placed close to the patient in circular mode at a radius of 24–25 cm.

#### Reconstruction

Data were reconstructed using a three-dimensional iterative method based on an ordered subset expectation maximization (3D-OSEM) algorithm with resolution recovery (RR; Flash 3D) and 120 updates (12 subsets × 10 iterations). This algorithm is available in the e.soft application package (Siemens). A Gaussian filter was used in the OSEM algorithm, and RC was used for smoothing. The full width at half maximum of the Gaussian filter was 13.2 mm.

#### Analysis and display

Gated SPECT images were automatically analyzed to calculate left ventricular (LV) volume and function, including end-diastolic volume (EDV), end-systolic volume (ESV), and left ventricular ejection fraction (LVEF), using quantitative gated SPECT (QGS) software (Cedars Sinai Medical Center, Los Angeles, CA, USA) and cardioREPO/EXINI Heart software. Myocardial perfusion defects were semi-quantified using a 17-segment model with 5-point visual scoring. The %uptake by each segment was analyzed using quantitative perfusion SPECT (QPS) software (Cedars Sinai Medical Center). We used normal databases from the Japanese Society of Nuclear Medicine (JSNM) Working Group, which was created by the collaboration of multiple Japanese institutions (Nakajima et al. 2007). The databases have been used in Japanese clinical practice, and the population-specific JSNM database was superior to American database (Nakajima et al. 2009). Ischemia was defined as a summed difference score between stress and resting images of two or more.

### Hybrid images of MP-SPECT and CCTA

Patients were assessed by CCTA using SOMATOM Definition Flash (Siemens Healthcare, Erlangen, Germany; Siemens Japan, Tokyo, Japan), a 128-section, dual X-ray source CT scanner. Images were acquired using CCTA after ~ 60 mL of contrast medium (Iopamidol 370 mg/mL, Iopamiron 370 mg/mL; Bayer Yakuhin, Ltd., Osaka, Japan) was administered at a flow rate of 4 mL/s followed by a 40 mL saline flush at the same rate. The scanning parameters were as follows: collimation, 128 × 0.6 mm; gantry rotation, 280 ms; step-and-shoot mode; tube voltage, 120 kV; tube current of 340 mAs/rotation; and a 220-mm field of view. Axial images were reconstructed with a slice thickness of 0.75 mm and a reconstruction kernel of B35f (Heart View medium). All data were transferred to an Advantage Workstation 4.6 (GE Healthcare, Chicago, IL, USA; GE Healthcare Japan, Tokyo, Japan) and then manual registration of the CCTA and MP-SPECT images proceeded using pixel-shifting.

### Artificial neural network

We used the computer-aided diagnostic software cardioREPO/EXINI Heart that was developed in Sweden and subsequently introduced into Japan (Nakajima et al. 2018). Version 1.1 of the artificial neural network (ANN) was trained on data derived from 1001 Japanese patients at 12 hospitals in Japan, and the diagnostic accuracy was validated in 364 patients (Nakajima et al. 2017). At least two Japanese nuclear cardiology specialist determined abnormal stress defects and stress-induced ischemia by consensus. Areas of possible perfusion abnormalities in stress and rest images (stress and rest defects, respectively) were segmented, and the ANN judged candidate regions based on 16 features extracted from the shape, extent, location, count, perfusion homogeneity, regional motion, wall thickening, and sex to give a probability of abnormality (ANN probability) (Nakajima et al. 2018). The cutoff value was defined as the ANN probability of 50%.

#### Image interpretation

**Fig. 1:**
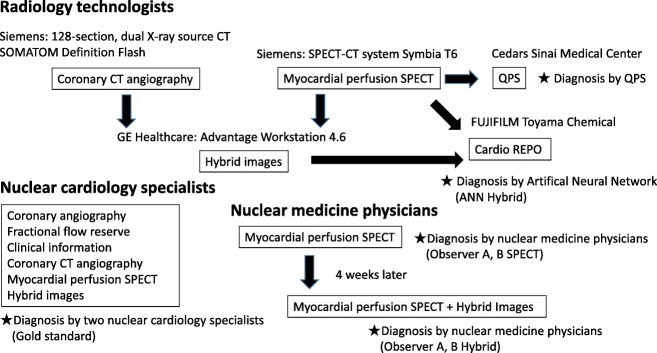
Basic flow chart of roles of radiology technologists, nuclear medicine physicians, and interpretation. *ANN* artificial neural network, *CT* computed tomography, *QPS* quantitative perfusion single-photon emission computed tomography, *SPECT* single-photon emission computed tomography

Figure 1 shows a flow chart of the image interpretation. A radiological technologist analyzed probabilities of perfusion abnormalities using the ANN and assigned the culprit coronary artery on hybrid images of CCTA and MP-SPECT. We defined the gold standard as the nuclear cardiology specialist interpretation with all available clinical information. Because patients with old myocardial infarction, percutaneous coronary intervention, and coronary artery bypass grafting were included, which is common in our clinical practice, the truth could not be simply defined by stenosis or fractional flow reserve (FFR). In addition, the AI was trained to learn specialist reading and not to identify possible stenosis or decreased flow reserve. The gold standard was, therefore, determined using coronary angiography and clinically available information including myocardial perfusion SPECT, CCTA, hybrid images, patient history, and subsequent clinical courses. Significant coronary artery disease was defined as stenosis in at least one coronary artery, as indicated by an FFR of 0.80 or less or stenosis diameter of > 70% using invasive coronary artery angiography or ischemia using hybrid images of CCTA and myocardial perfusion SPECT if FFR was not available. Ischemia using hybrid images of CCTA and myocardial perfusion SPECT was defined by inducible perfusion abnormality judged by two nuclear cardiology specialists. Two other nuclear medicine physicians evaluated the SPECT images, then the hybrid images of CCTA and MP-SPECT in the same way 4 weeks later, using four grades of confidence to evaluate their interpretation of regional abnormalities as follows: 1, definitely normal; 2, probably normal; 3, probably abnormal; and 4, definitely abnormal. This cutoff value was defined as 3. The same physicians then drew the regions on polar maps and evaluated confidence in their interpretations of the culprit coronary artery as RCA, LCX, left anterior descending coronary artery (LAD), RCA or LCX (equivocal), LCX or LAD (equivocal), and LAD or RCA (equivocal). Diagnostic performance, including sensitivity, specificity, and accuracy in the detection of culprit coronary arteries, was compared among observer A, observer B, and the ANN.

### Statistical analysis

Data were statistically analyzed using JMP version 12 (SAS Institute Inc., Cary, NC, USA). We evaluated CAD detectability based on areas under receiver operating characteristic (ROC) curves (AUC). All statistical tests were two-tailed, and values with *p* <  0.05 were considered statistically significant.

## Results

### Patient-based diagnosis

**Fig. 2:**
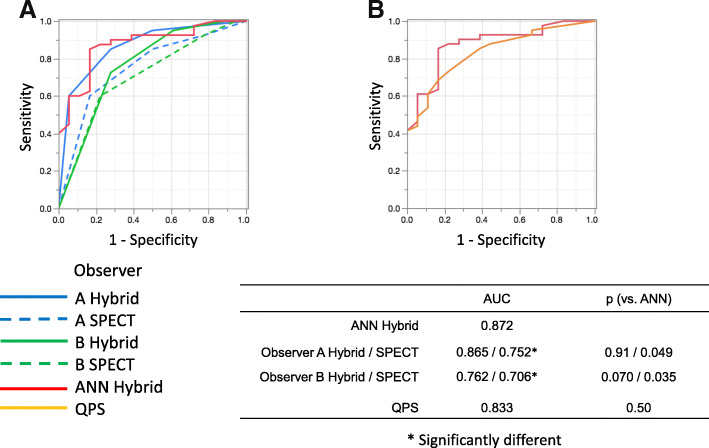
Areas under ROC curves derived from patient-based analysis by ANN, two observers (**a**), and QPS (**b**). *ANN* artificial neural network, *QPS* quantitative perfusion SPECT, *ROC* receiver operating characteristics

Table 2 shows the likelihood ratios, Pearson statistics, and *p* values for observers A, B, and the ANN. The abilities of the ANN and nuclear medicine physicians to diagnose infarction and/or ischemia were equivalent, and the ANN outperformed conventional semi-quantitative defect scoring using QPS (Fig. 2). The sensitivity, specificity, and accuracy of patient-based diagnoses by observers A and B using hybrid images were 85.0%, 72.2%, and 81.0% and 73.1%, 73.2%, and 78.0%, respectively, and these values for the ANN and QPS were 87.8%, 72.2%, and 84.7%, and 73.2%, 77.8%, and 74.6%, respectively.

**Table 2:** Contingency analysis of ANN and two observers

	True findings				
	Infarction	Infarction + Ischemia	Ischemia	None	Total
A. Observer A					
Infarction	10	4	1	1	16
Infarction + Ischemia	2	4	6	0	12
Ischemia	0	1	6	4	11
None	3	2	2	13	20
Total	15	11	15	18	59
B. Observer B					
Infarction	9	3	2	3	17
Infarction + Ischemia	5	6	7	5	23
Ischemia	1	1	5	3	10
None	0	1	1	7	9
Total	15	11	15	18	59
C. ANN					
Infarction	10	0	1	2	13
Infarction + Ischemia	3	9	5	0	17
Ischemia	0	0	7	1	8
None	2	2	2	15	21
Total	15	11	15	18	59
D. Statistical significance					
	Likelihood ratio	*p* value	Pearson statistics	*p* value	
ANN	65.6	< 0.0001	68.1	< 0.0001	
Observer A	44.8	< 0.0001	41.1	< 0.0001	
Observer B	22.1	0.0087	22.4	0.0076	

The likelihood ratios, Pearson statistics, and *p* values for observer A (A), observer B (B), and ANN (C)

Likelihood ratios, Pearson statistics, and probability (*p*) estimates for observers A, B, and artificial neural network (ANN)

### Coronary artery-based diagnosis

**Fig. 3:**
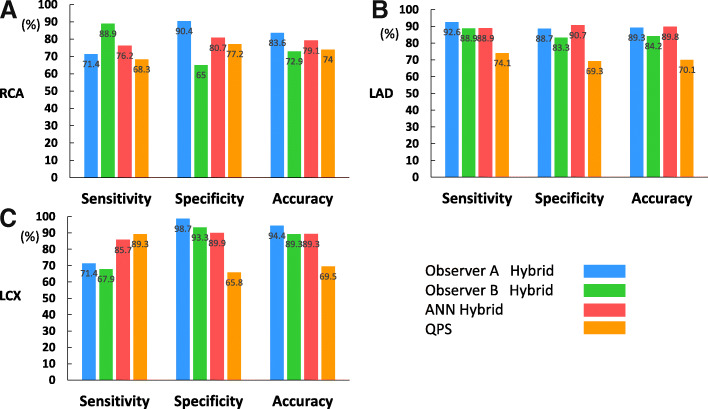
Sensitivity, specificity, and accuracy of CAD detection in RCA (**a**), LAD (**b**), and LCX (**c**), by ANN, QPS, and two observers with hybrid images. *ANN* artificial neural network, *CAD* coronary artery disease, *LAD* left anterior descending coronary artery, *LCX* left circumflex coronary artery, *QPS* quantitative perfusion single-photon emission computed tomography (SPECT), *RCA* right coronary artery

**Fig. 4:**
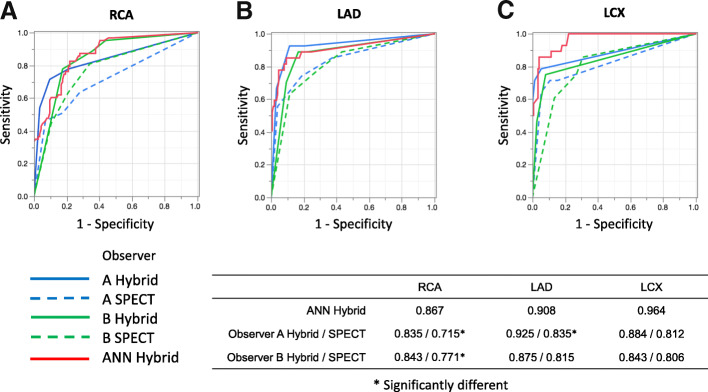
Areas under ROC curves derived from analysis of RCA (**a**), LAD (**b**), and LCX (**c**) by ANN and two observers using hybrid images and SPECT. *ANN* artificial neural network, *LAD* left anterior descending coronary artery, *LCX* left circumflex coronary artery, *RCA* right coronary artery, *ROC* receiver operating characteristic, *SPECT* single-photon emission computed tomography

**Fig. 5:**
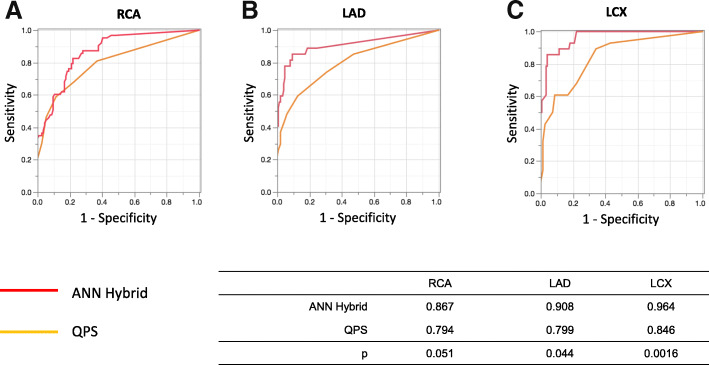
Areas under ROC curves derived from analysis of RCA (**a**), LAD (**b**), and LCX (**c**) by ANN and QPS. *ANN* artificial neural network, *LAD* left anterior descending coronary artery, *LCX* left circumflex coronary artery, *QPS* quantitative perfusion single-photon emission computed tomography, *RCA* right coronary artery, *ROC* receiver operating characteristics

**Fig. 6:**
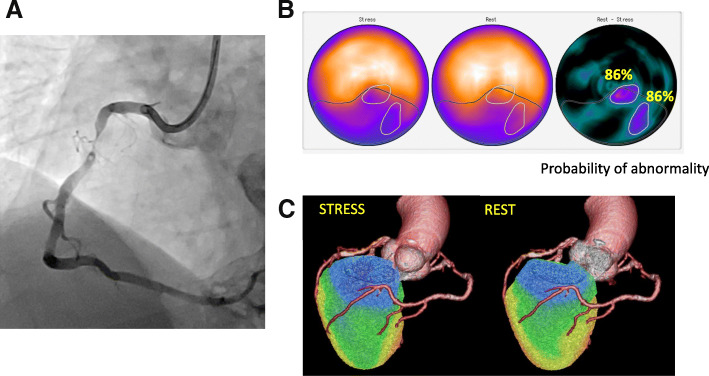
Highest stress defect score and ischemia determined by ANN and two observers in 79-year-old man with angina pectoris. Coronary angiography revealed 90% stenosis in RCA (**a**). Consensus interpretation diagnosed stress-induced ischemia and infarct in the inferior walls. Defect SSS, SRS, and SDS were 11, 6, and 5, respectively. Artificial neural network detected abnormality in inferior regions under stress-induced ischemia (black contour, ANN probability: 0.97) and ischemia (white contour, ANN probability: 0.86) images (**b**). Hybrid images of CCTA and myocardial perfusion SPECT identify culprit as RCA (**c**). *ANN* artificial neural network, *CAG* coronary angiography, *LAD* left anterior descending coronary artery, *RCA* right coronary artery, *SDS* summed difference score, *SRS* summed rest score, *SSS* summed stress score

Figure 3 shows the sensitivity, specificity, and accuracy of coronary-based diagnosis by observer A, observer B, the ANN, and QPS in identifying culprits among the RCA, LAD, and LCX. Figures 4 and 5 show the AUC of observer A, observer B, the ANN, and QPS. The AUC were significantly improved using hybrid images in the RCA region (observer A, *p* = 0.0031; observer B, *p* = 0.042). The culprit was most accurately diagnosed in the LAD region. The AUC to detect RCA lesions were better for CTA-guided myocardial perfusion than MP-SPECT image analysis. Figure 6 shows a patient with single-vessel disease of the RCA in which the ANN system interpreted the images of a detected ischemic area.

### Anatomical reference-based analysis

**Fig. 7:**
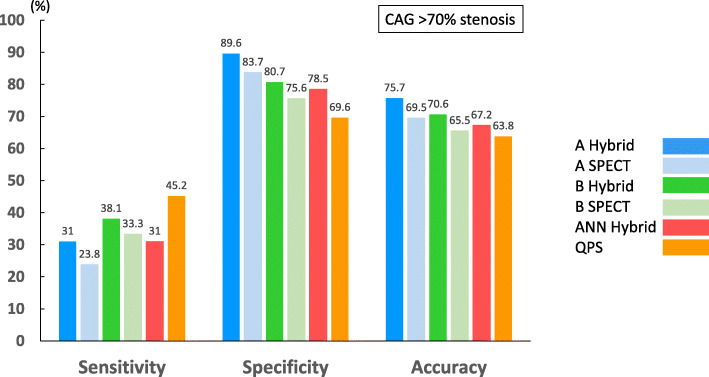
Sensitivity, specificity, and accuracy of coronary artery-based diagnosis by observer A, observer B, ANN, and QPS in patients with myocardial perfusion SPECT and hybrid images when compared to anatomical reference standard defined as a stenosis diameter of > 70% by invasive coronary angiography. *ANN* artificial neural network, *QPS* quantitative perfusion single-photon emission computed tomography

Figure 7 showed the comparison of coronary artery-based diagnosis by observer A, observer B, ANN, and QPS in patients with MP-SPECT and hybrid images of CCTA and MP-SPECT when compared against anatomical reference standard defined as a stenosis diameter of > 70% by invasive coronary angiography. The AUC of observers A and B using hybrid images were 0.606 and 0.584 and using MP-SPECT were 0.576 and 0.546, respectively. The AUC was not significantly improved using hybrid images. The AUC of ANN and QPS were 0.568 and 0.544, respectively. The AUC was not significantly improved using ANN.

## Discussion

This study demonstrated that hybrid images of CCTA and MP-SPECT with neural network guidance could identify perfusion abnormalities effectively. When a radiology technologist analyzed hybrid images and MP-SPECT images using AI-based software to identify a culprit coronary artery, the diagnostic ability of the ANN was comparable to that of nuclear medicine physicians and outperformed conventional semi-quantitative defect scoring.

### The need for applying AI software

MPI has been interpreted based on integrated understanding of myocardial perfusion distribution at stress and rest, and comparison between these two conditions using visual analysis and automatic scoring contributed to diagnosis of ischemia in clinical practice. However, while typical perfusion defects or ischemia could be unanimously judged as abnormal, nuclear physicians sometimes struggle to diagnose for equivocal findings; in such situation, a method to support objective and quantitative interpretation is considered useful. Nuclear medicine physicians tend to intentionally overlook or over-read MPI findings based on the experience of previous success or failure of interpretation. On the other hand, the ANN could emphasize possible abnormal regions with sufficient degrees of certainty and reproducibility. Since cardiologists and radiologists are not always specialists in nuclear cardiology, such ANN-based suggestions might enhance diagnostic confidence. The probability of abnormalities provided by the ANN might be useful reference even for nuclear cardiology specialist. When a radiology technologist analyzed hybrid images and MP-SPECT images using AI-based software to identify a culprit coronary artery, the diagnostic ability of the ANN was comparable to that of nuclear medicine physicians and outperformed conventional semi-quantitative defect scoring. This finding was consistent with those of other studies (Nakajima et al. 2018; Nakajima et al. 2017; Nakajima et al. 2015; Johansson et al. 2014).

### Identification of coronary artery lesions

Our analysis of diagnostic performance in specific vessels showed that CCTA-guided quantitation was useful in the RCA territory, and that the diagnostic accuracy was comparable in the LAD territory between nuclear physicians and software-guided technologist interpretation. Clinicians must accurately match the anatomy of the coronary artery with the location of an abnormality in SPECT images to accurately assess myocardial ischemia. Generally, the LCX flows orthogonally to the LAD from the 11 o’clock position in polar maps. The obtuse marginal and posterolateral branches at 2–3 and 4–5 o’clock respectively emerge from the LCX in the left atrioventricular groove. The RCA runs in the right atrioventricular groove and sends out the posterior descending artery at 7 o’clock. The atrioventricular node artery branches from the posterior descending artery toward the 5–6 o’clock region (Nagano et al. 2016). However, using only SPECT to assign coronary artery territories is difficult because perfused regions of the LCX and RCA vary among individuals. Therefore, CCTA is needed to accurately identify culprit coronary arteries in patients with ischemia.

### Accuracy of MP-SPECT to detect coronary artery disease

Nakajima et al. reported that the sensitivity, specificity, and accuracy of interpreting culprit coronary artery territories using MP-SPECT without hybrid images were of 77%, 74%, and 75%, respectively (Nakajima et al. 2010). Quantitative analysis based on summed QPS stress scores of ≥ 4 was used as threshold of abnormality; Johansson et al. reported that the specificity of computer-aided diagnostic software was higher than that of summed stress scores (86.8% vs. 73.6%; *p* <  0.001) at the same level of sensitivity. Danad et al. reported that quantitative analysis based on summed QPS difference scores ≥ 2 conferred an overall sensitivity, specificity, and accuracy of hybrid SPECT and CCTA for diagnosis of ischemia-causing coronary artery disease as indicated by FFR were 50%, 97%, and 76% (Danad et al. 2017). Although the diagnostic ability was dependent on the patient selection criteria, these results of diagnostic ability were comparable to our finding and support our conclusions.

### Reduction of artifacts

Since the ANN does not consider body motion, body attenuation, or artifacts, these factors need to be reduced. Body motion tends to result in misdiagnosis, particularly when using the ANN. Although this can be addressed using motion correction software, reacquisition should proceed when large amounts of motion are detected by linography and/or sinography. Activity in sub-diaphragmatic organs can cause artifacts in images by simulating perfusion defects and concealing true defects. Iterative reconstruction suppresses streak artifacts caused by high extracardiac activity such as gallbladder activity. Reacquisition should also be considered when the extracardiac uptake of ^99m^Tc is more intense than cardiac uptake, or when extracardiac uptake is not distinguishable from the inferior cardiac wall. Importantly, the ANN might not detect widespread, very mild ischemia in poor-quality images. Less noisy images are generally expected, even with limited radiation doses. Thus, dose and acquisition time should be balanced.

### Disadvantages of hybrid images of CCTA and MP-SPECT

Although hybrid images of CCTA and MP-SPECT improves the detection of coronary artery disease and enhances diagnostic confidence, this procedure has not yet become routine in the clinical setting at many hospitals. The hybrid images of CCTA and MP-SPECT can require time-consuming manual intervention (Piccinelli et al. 2018). Radiation doses delivered to patients should also be considered. Coronary arteries can be directly visualized by CCTA and coronary atherosclerosis can be detected and quantified. Individual coronary plaques can be identified, and information can be gathered regarding the total extent, severity, location, and composition of coronary artery disease (van Rosendael et al. 2017). However, a phantom study found that the radiation dose of 128-section, dual-source CT coronary angiography was 195.7 mGy in the low-pitch spiral mode and 96.1 mGy in the step-and-shoot mode (Matsubara et al. 2012). Thus, the latter mode should initially be applied because less radiation is absorbed.

### Study limitations

This single-center, retrospective, observational study has several limitations. We did not measure FFR in all patients and the cohort of patients was quite small. It was difficult to measure FFR in all cases and all stenotic coronary arteries. Therefore, data selection was biased by the clinical conditions that prompted the need for invasive angiography, SPECT, and CCTA, as only a minority of patients are evaluated for CAD using these modalities.

We did not apply fully automated quantitative analysis or image registration; the contour definitions and vascular territories were manually guided by co-registered CCTA anatomy.

## Conclusions

Hybrid images of CCTA and MP-SPECT improved the ability to identify culprit coronary arteries in patients with stable ischemic heart disease. The diagnostic abilities of the ANN and nuclear medicine physicians were comparable. Because of interindividual differences in the perfused regions of the RCA and LCX, to identify a coronary artery responsible for perfusion defects of the inferior wall is difficult without using hybrid images.

## Abbreviations

3D-OSEM: Three-dimensional iterative method based on ordered subset expectation maximization
AI: Artificial intelligence
ANN: Artificial neural network
AUC: Area under ROC curve
CAD: Coronary artery disease
CCTA: Coronary computed tomography angiography
ECG: Electrocardiography
EDV: End-diastolic volume
ESV: End-systolic volume
FFR: Fractional flow reserve
LAD: Left anterior descending coronary artery
LCX: Left circumflex coronary artery
LV: Left ventricular
LVEF: Left ventricular ejection fraction
MPI: Myocardial perfusion imaging
QGS: Quantitative gated SPECT
QPS: Quantitative perfusion SPECT
RCA: Right coronary artery
ROC: Receiver operating characteristics
RR: Resolution recovery
SPECT: Single-photon emission computed tomography
